# Prevalence and Correlates of Caffeine Use Disorder Symptoms Among a United States Sample

**DOI:** 10.1089/caff.2019.0020

**Published:** 2020-03-04

**Authors:** Mary M. Sweeney, Darian C. Weaver, Kathryn B. Vincent, Amelia M. Arria, Roland R. Griffiths

**Affiliations:** ^1^ Behavioral Pharmacology Research Unit, Department of Psychiatry and Behavioral Sciences, Johns Hopkins University School of Medicine, Baltimore, Maryland.; ^2^ Center on Young Adult Health and Development, Department of Behavioral and Community Health, University of Maryland School of Public Health, College Park, Maryland.; ^3^ Department of Neuroscience, Johns Hopkins University School of Medicine, Baltimore, Maryland.

**Keywords:** caffeine use disorder, caffeine dependence, caffeine withdrawal, tobacco, sleep, anxiety

## Abstract

***Background:*** The *DSM-5* recognizes caffeine use disorder as a condition for further study, but there is a need to better understand its prevalence and clinical significance among the general population.

***Methods:*** A survey was conducted among an online sample of 1006 caffeine-consuming adults using demographic quotas to reflect the U.S. population. Caffeine consumption, *DSM*-proposed criteria for caffeine use disorder, sleep, substance use, and psychological distress were assessed.

***Results:*** Eight percent of the sample fulfilled *DSM*-proposed criteria for caffeine use disorder. These individuals consumed more caffeine, were younger, and were more likely to be cigarette smokers. Fulfilling caffeine use disorder criteria was associated with caffeine-related functional impairment, poorer sleep, some substance use, as well as greater depression, anxiety, and stress.

***Conclusions:*** The prevalence of caffeine use disorder among the present sample suggests that the proposed diagnostic criteria would identify only a modest percentage of the general population, and that identified individuals experience significant caffeine-related distress.

## Introduction

Caffeine is a commonly consumed psychoactive drug which produces its psychomotor stimulant and reinforcing effects through antagonism at adenosine receptors and indirect effects on dopaminergic neurotransmission.^1^ Consumption of caffeine at recommended dietary doses is not generally associated with negative health consequences,^2^ and caffeine has some clinical utility, such as for enhancement of analgesia.^3^ However, there is also evidence that some caffeine consumers might experience caffeine-related health effects and functional impairment. Higher doses of caffeine can produce dysphoric subjective effects and caffeine intoxication, including restlessness, nervousness, insomnia, gastrointestinal distress, and irregular heartbeat.^4–7^ Caffeine is contraindicated for gastrointestinal problems, urinary incontinence, insomnia, and anxiety, and use during pregnancy is associated with poor outcomes.^8,9^ After discontinuing regular use, some individuals experience withdrawal symptoms, including headache, fatigue, irritability, depressed mood, difficulty concentrating, and flu-like symptoms.^10,11^ *DSM-5* and ICD-10 recognize caffeine intoxication, caffeine withdrawal, caffeine-induced anxiety disorder, and caffeine-induced insomnia as potential diagnoses when symptoms cause clinically significant distress or impairment.^12,13^ Because some individuals report an inability to cut down or reduce their caffeine consumption despite clinically significant problems caused or exacerbated by continued caffeine consumption and seek treatment for their caffeine consumption,^14^ substance dependence due to caffeine is included in ICD-10, and caffeine use disorder was included in *DSM-5* in section III as a condition for further study.^12,13^

*DSM-5* proposed three necessary and sufficient diagnostic criteria for caffeine use disorder: (1) a persistent desire or unsuccessful efforts to cut down or control caffeine use; (2) continued caffeine use despite knowledge of having a persistent or recurrent physical or psychological problem that is likely to have been caused or exacerbated by caffeine; and (3) withdrawal, as manifested by the characteristic withdrawal syndrome for caffeine, or caffeine or a closely related substance is taken to relieve or avoid withdrawal symptoms. Six additional diagnostic criteria included in other substance use disorders, such as craving, tolerance, and taking caffeine in larger amounts or over a longer period of time than intended, were also included as markers for greater severity beyond the three key criteria for caffeine use disorder. To mitigate the potential for overdiagnosis given the ubiquity of caffeine consumption, the proposed diagnostic strategy for caffeine is more conservative than for other substances, which require fulfillment of any 2 of 11 diagnostic symptoms to meet criteria for mild substance use disorder.^14,15^

Lack of data regarding the prevalence and clinical significance of caffeine use disorder among general population samples was cited as the reason for its inclusion in *DSM-5* as a disorder for further study rather than as a recognized diagnosis.^13^ Studies have examined the prevalence of substance use disorder criteria as applied to caffeine, but the majority were conducted among special populations such as heavy or treatment-seeking caffeine consumers or psychiatric patients, they preceded the proposed *DSM-5* criteria, or had relatively small sample sizes.^6,16–27^ The only general population examination of *DSM*-defined caffeine use disorder in the United States surveyed 162 current caffeine consumers in Vermont and found that 30% of caffeine consumers met generic *DSM-IV* criteria for substance dependence as applied to caffeine.^6^ The estimated prevalence was less than 10% when a key-criteria strategy similar to that proposed by *DSM-5* was adopted. A prevalence of *DSM-5*-defined caffeine use disorder among a nonclinical sample much higher than previous estimates would support the concern for overdiagnosis. Further, the extent to which caffeine use disorder is associated with markers of clinical significance such as self-reported caffeine-related distress or impairment, psychological distress, sleep problems, or other drug use is unknown. The present study aimed to examine the prevalence and correlates of *DSM-5*-defined problematic caffeine use among a large sample of U.S. adults.

## Methods

### Participants

Data were collected anonymously using Qualtrics Research Services, an online survey panel aggregator, which has been utilized in other peer-reviewed research studies.^28–31^ Participants were recruited from 21 actively managed online research panels with more than 13.4 million registered panelists. Recruitment quotas were based on U.S. census data to reflect the age, sex, race, and ethnicity of the general U.S. population.

The first page of the survey described the questions and research purpose, risks, and noted that by continuing, participants affirm that they are 18 years or older, speak English fluently, reside in the United States, and voluntarily agree to participate. It was stated that completion of the survey served as consent and if stopped early, responses would not be used. One hundred eighty-four respondents entered the survey but did not consent (exited survey), and 125 consented and began the survey but did not complete it. Exclusionary criteria were as follows: (1) no caffeine consumption during the past 12 months (92 excluded), (2) no caffeine consumption during a typical week (23 excluded), (3) indicating 17 years or younger in demographic questions (25 excluded), (4) irrelevant or gibberish responses in open-ended text responses (94 excluded), and (5) speeding, that is, survey completion time less than one-third of the median completion duration during initial testing (15 excluded). The final sample included 1006 participants. The Johns Hopkins University Institutional Review Board determined that the present study was exempt research.

### Measures

#### Demographic information

Age, sex (assigned at birth and current sex or gender), ethnicity, and race were collected using standard questions.

#### Caffeine consumption

Participants selected caffeinated products consumed during a typical week from a list (i.e., coffee, tea, soft drinks, energy drinks, energy shots, caffeine-containing medicines/supplements). For each selection, participants reported typical serving size in ounces (or tablet/capsules), typical number of servings on a day they consume the product, and how many days per week each product was typically consumed. Participants were asked to exclude decaffeinated items. Milligrams of caffeine per serving was calculated using typical milligrams per ounce for brewed/drip coffee (200 mg/12 oz), brewed tea (40 mg/6 oz), and soft drinks (40 mg/12 oz).^9^ For energy drinks, energy shots, and caffeine-containing medicines, caffeine per serving was calculated using each individual's most commonly used brand. Total caffeine intake per week from all sources was summed and divided by seven to estimate daily caffeine consumption. We calculated the proportion of weekly caffeine intake from the various sources of caffeine for each individual.

#### Caffeine-related problems

Caffeine use disorder symptoms were assessed using *DSM-5* criteria for substance use disorder as applied to caffeine based on structured clinical interviews administered in prior clinical trials,^26,32^ in which participants indicated whether or not and to what extent each symptom was experienced during the past 12 months. To meet individual criteria, participants must have indicated its occurrence during the past 12 months with sufficient frequency or intensity.^26,32^ Participants were asked to rate on a scale from 0 to 10 (where 0 is not present and 10 is extreme), how much overall distress they experienced due to their caffeine consumption during the past 12 months. Participants were also asked, “During the past 12 months, have you felt bad or guilty about your caffeine consumption?” (Yes or No). If they indicated “Yes,” they were asked how often this occurred (Daily, Weekly, Monthly, A few times during the past 12 months, or Once during the past 12 months). If participants indicated physical problems, psychological problems, or withdrawal symptoms related to caffeine, they were asked to individually rate from 0 to 10 (where 0 is not present and 10 is extreme), how much the physical problems, psychological problems, or withdrawal symptoms disrupted their ability to function in their life at work, school, or at home during the past 12 months.

#### Substance use

Participants reported current combustible tobacco and e-cigarette use. Alcohol consumption was assessed by asking days of use during the past 30 days. Similarly, past 30-day drug use (i.e., cannabis-medical, cannabis-recreational, cocaine, inhalants, hallucinogens, heroin, amphetamines, methamphetamines, and 3,4-methylenedioxymethamphetamine (MDMA)/ecstasy) and nonmedical prescription drug use (i.e., stimulants, opioid analgesics, tranquilizers, and sedatives) were measured. The Alcohol Use Disorders Identification Test-Concise (AUDIT-C) assessed risk for alcohol dependence.^33^ Participants reported whether or not they had ever received treatment for substance use.

#### Sleep and psychological distress

Eight items from the Pittsburgh Sleep Quality Index^34^ assessed sleep latency, sleep duration, and frequency of problems such as waking in the night. Scores ranged 0–24 where higher scores indicate greater problems. Participants completed the Depression Anxiety and Stress Scales-21 (DASS-21)^35^ as an index of psychological distress.

### Statistical analysis

Basic demographic and caffeine consumption information was summarized for the overall sample, as was the prevalence of meeting *DSM-5*-proposed criteria for caffeine use disorder. Multivariable logistic regression was used to estimate the strength of the association between age, sex (assigned at birth), minority status (i.e., dichotomous white non-Hispanic or other race/ethnicity), current combustible tobacco use, and estimated daily caffeine consumption and meeting all three key *DSM-5*-proposed criteria for caffeine use disorder (yes/no). Estimated daily caffeine consumption data were log transformed to correct positive skew. Individuals who did and did not meet caffeine use disorder criteria were compared on self-reported caffeine-related distress and functional impairment, drug and alcohol use, sleep problems, depression, anxiety, and stress while controlling for age, sex, minority status, and current combustible tobacco use using multivariable linear regression or logistic regression. Substance use, ratings of frequency of feeling bad or guilty about caffeine consumption, impairment due to physical problems, psychological problems, and withdrawal were dichotomized due to low overall prevalence. For each individual, the number of *DSM-5* criteria met (0–11) was totaled, and this variable was examined in relationship to the variables described above.

## Results

### Demographics and caffeine intake

Demographic information is shown in Table 1. The final sample (*n* = 1006) was 62% female, 73% white non-Hispanic, and the mean age was 47.4 years (SD = 16.4). Coffee was the most common source of caffeine; 73% of participants consumed coffee during a typical week, followed by soft drinks (64%), tea (43%), energy drinks (18%), caffeine-containing medicines/supplements (10%), and energy shots (6%). The majority of participants (67%) consumed caffeine from more than one source. Coffee accounted for the greatest mean proportion of weekly caffeine consumption (*M* = 0.57, SD = 0.40), followed by soft drinks (*M* = 0.20, SD = 0.32) and tea (*M =* 0.15, SD = 0.28). Mean percentage of weekly caffeine consumption was less than 10% for other sources. Median estimated daily caffeine intake from all sources was 292 mg (*M* = 506.9, SD = 747.7) for the overall sample.

**Table 1.** Demographic Characteristics of the Overall Sample (*n* = 1006)

Characteristic	
Age, mean (SD)	47.4 (16.4)
Sex assigned at birth, count (%)^a^	
Female	619 (62)
Male	386 (38)
Intersex	1 (<1)
Race, count (%)	
White	784 (78)
Black or African American	129 (13)
Asian	49 (5)
More than one race	20 (2)
Native Hawaiian or other Pacific Islander	6 (1)
American Indian/Alaska Native	5 (<1)
Other	13 (1)
Ethnicity, count (%)	
Non-Hispanic	926 (92)
Hispanic	80 (8)
Substance use, count (%)	
Current combustible tobacco smoker	311 (31)
Current e-cigarette user	127 (13)
Past 30-day alcohol use	641 (64)
Past 30-day cannabis use	156 (16)
Past 30-day illicit substance use other than cannabis	43 (4)
Past 30-day nonmedical use of prescription drugs	104 (10)
Ever treated for substance use	69 (7)

^a^ The intersex participant was included as female because they identified as female and trans male when indicating current gender.

SD, standard deviation.

### Prevalence of diagnostic criteria

Prevalence of meeting diagnostic criteria for caffeine use disorder is shown in Table 2. Eight percent of the sample (*n* = 84) fulfilled all three key diagnostic criteria for caffeine use disorder as proposed by *DSM-5*, including a persistent desire or unsuccessful efforts to reduce caffeine use (34% prevalence), continued caffeine use despite a physical or psychological problem likely to have been caused or exacerbated by caffeine use (17%), and caffeine withdrawal (27%), as manifested by either the characteristic withdrawal syndrome for caffeine (16%) or for taking caffeine to relieve or avoid withdrawal symptoms (23%). Among those who met the three key criteria for caffeine use disorder, the three most common withdrawal symptoms were headache (79%), fatigue (42%), and irritability (36%); the most common psychological problem caused or worsened by caffeine was anxiety (25%), and the most common physical problem was sleep disturbance (33%). Individuals who were younger, consumed more caffeine, and current combustible tobacco users were more likely to meet all three *DSM-5*-proposed criteria for caffeine use disorder (Table 3), whereas sex and minority status were not significant correlates.

**Table 2.** Prevalence of *DSM-5* Caffeine Use Disorder Criteria for the Overall Sample (*n* = 1006)

Criterion	n	%
1. A persistent desire or unsuccessful efforts to cut down or control caffeine use.	340	34
2. Continued caffeine use despite knowledge of having a persistent or recurrent physical or psychological problem that is likely to have been caused or exacerbated by caffeine.	168	17
3. Withdrawal, as manifested by either of the following:	270	27
a. The characteristic withdrawal syndrome for caffeine.	165	16
b. Caffeine (or a closely related substance) is taken to relieve or avoid withdrawal symptoms.	230	23
4. Caffeine is often taken in larger amounts or over a longer period than was intended.	506	50
5. Recurrent caffeine use resulting in a failure to fulfill major role obligations at work, school, or home.	77	8
6. Continued caffeine use despite having persistent or recurrent social or interpersonal problems caused or exacerbated by the effects of caffeine (e.g., arguments with spouse about consequences of use, medical problems, cost).	33	3
7. Tolerance, as defined by either of the following:	277	28
a. A need for markedly increased amounts of caffeine to achieve desired effect.	146	15
b. Markedly diminished effect with continued use of the same amount of caffeine.	248	25
8. A great deal of time is spent in activities necessary to obtain caffeine, use caffeine, or recover from its effects.	339	34
9. Craving or a strong desire or urge to use caffeine.	108	11
10. Important social, occupational, or recreational activities are given up or reduced because of caffeine use.	129	13
11. Recurrent caffeine use in situations in which it is physically hazardous.	49	5
*Mild:* Met two or three criteria	238	24
*Moderate:* Met four or five criteria	144	14
*Severe:* Met six or more criteria	115	11
*DSM-5-proposed caffeine use disorder:* Met at least criteria 1, 2, and 3 and above	84	8

**Table 3.** Multivariable Logistic Regression Evaluating Demographic Correlates of Meeting Key Criteria for Caffeine Use Disorder

Correlate	B	SE	OR	95% CI	
				Lower	Upper
Estimated daily caffeine intake^a^	0.89	0.25	2.43	1.48	3.99
Age in years^a^	−0.03	0.01	0.97	0.95	0.98
Current combustible tobacco use^a^	0.52	0.25	1.68	1.04	2.71
Minority status	0.29	0.26	1.34	0.80	2.23
Sex	−0.34	0.27	0.71	0.42	1.22
χ^2^	45.6^a^				
df	5				
Nagelkerke R^2^	0.10				

*Note*. Caffeine intake (mg) was log-transformed; Reference categories are nonsmoker (combustible tobacco use), white non-Hispanic (minority status) and female (sex).

^a^ Indicates *p* < 0.05 statistical significance.

CI, confidence interval; OR, odds ratio; SE, standard error.

### Caffeine-related distress

Meeting the proposed key criteria for caffeine use disorder was significantly associated with caffeine-related distress, feeling bad or guilty about caffeine use, functional impairment due to caffeine withdrawal symptoms, psychological problems caused or worsened by caffeine, and physical problems caused or worsened by caffeine after controlling for age, sex, minority status, and tobacco use (Table 4; left columns). Regression analyses showed that the more caffeine use disorder criteria met, the higher the caffeine-related distress, the greater the likelihood of feeling bad or guilty about caffeine use, and the greater the likelihood of caffeine-related functional impairment (Table 4; right columns).

**Table 4.** Multivariable Regression Analyses Evaluating Caffeine Use Disorder Criteria as Correlates of Caffeine-Related Distress, Health and Substance Use Variables, Controlling for Demographic Variables

Variable	CUD present as correlate						Number of CUD criteria as correlate					
	B	SE	β	OR	95% CI		B	SE	β	OR	95% CI	
					Lower	Upper					Lower	Upper
Caffeine-related distress/impairment												
Overall caffeine-related distress (0–10 scale)	2.92	0.24	0.35^a^		2.45	3.40	0.56	0.03	0.58^a^		0.51	0.62
Feeling bad or guilty about caffeine use	2.53	0.27		12.58^a^	7.46	21.22	0.54	0.05		1.71^a^	1.57	1.87
Impairment due to psychological problem	2.46	0.26		11.65^a^	7.02	19.32	0.54	0.05		1.71^a^	1.55	1.88
Impairment due to physical problem	2.73	0.27		15.31^a^	9.10	25.77	0.50	0.04		1.64^a^	1.51	1.79
Impairment due to caffeine withdrawal	3.57	0.44		35.49^a^	15.00	83.98	0.63	0.05		1.88^a^	1.72	2.06
Sleep problems (0–24 scale)	5.17	0.55	0.28^a^		4.09	6.25	0.95	0.07	0.45^a^		0.82	1.08
DASS-21												
Depression (0–21 scale)	3.94	0.57	0.21^a^		2.83	5.06	0.86	0.07	0.40^a^		0.73	0.99
Anxiety (0–21 scale)	4.69	0.45	0.30^a^		3.80	5.58	0.94	0.05	0.52^a^		0.84	1.04
Stress (0–21 scale)	4.56	0.50	0.26^a^		3.58	5.53	0.92	0.06	0.46^a^		0.80	1.03
Other substance-related variables												
AUDIT-C (0–12 scale)	0.38	0.29	0.04		−0.19	0.96	0.12	0.04	0.11^a^		0.05	0.19
Past 30-day substance use												
Cocaine	1.56	0.59		4.76^a^	1.50	15.08	0.28	0.09		1.32^a^	1.10	1.58
Heroin	2.26	1.06		9.58^a^	1.20	76.39	0.33	0.17		1.39	1.00	1.93
Inhalants	3.30	1.62		27.23^a^	1.14	651.27	0.54	0.26		1.71^a^	1.02	2.87
Amphetamines	0.69	0.60		1.99	0.61	6.42	0.26	0.08		1.30^a^	1.10	1.53
Methamphetamines	0.49	0.83		1.62	0.32	8.22	0.30	0.11		1.35^a^	1.09	1.68
Nonmedical use of prescription stimulants	0.91	0.49		2.48	0.96	6.45	0.19	0.07		1.20^a^	1.04	1.39
Nonmedical use of prescription opioids	0.39	0.46		1.48	0.60	3.67	0.26	0.06		1.29^a^	1.16	1.44
Nonmedical use of prescription tranquilizers	0.47	0.44		1.60	0.68	3.78	0.20	0.06		1.22^a^	1.09	1.36
Nonmedical use of prescription sedatives	0.91	0.53		2.49	0.88	7.01	0.32	0.07		1.37^a^	1.19	1.59
Ever treated for substance use	0.62	0.36		1.86	0.91	3.80	0.18	0.05		1.20^a^	1.09	1.32

*Note*. Each variable row indicates an individual regression analysis which controlled for age, sex, minority status, and current combustible tobacco use. Left columns describe regression analyses where dichotomous CUD status (all three key criteria present vs. absent) was a correlate, whereas right columns describe regression analyses where the number of CUD criteria met (0–11 criteria) was a correlate. Scale is specified for variables evaluated with linear regression, otherwise variables were dichotomized (present vs. absent) and analyzed using logistic regression where absent is the reference category. Past 30-day substance use for alcohol, cannabis, hallucinogens, and ecstasy are not included in the table as they were not significant.

AUDIT-C, Alcohol Use Disorders Identification Test-Concise; CUD, caffeine use disorder; DASS, Depression, Anxiety, and Stress Scale.

^a^ Indicates *p* < 0.05 statistical significance.

### Sleep and psychological distress

Meeting key diagnostic criteria for caffeine use disorder was associated with total sleep problems and psychological distress (Table 4; left columns). Meeting a greater number of criteria was also significantly associated with greater sleep problems (Table 4; right columns). Meeting key criteria for caffeine use disorder and meeting a greater number of criteria were both associated with higher scores for all three subscales of the DASS-21 indicating greater depression, anxiety, and stress (Table 4).

### Substance use

Meeting criteria for caffeine use disorder was significantly associated with some, but not all, substance use other than caffeine. Table 4 displays the regression results for only the substance use variables for which either meeting key criteria for caffeine use disorder (left columns) or the number of caffeine use disorder criteria met (0–11; right columns) were significantly associated with substance use. Meeting key criteria was significantly associated with past 30-day use of cocaine, inhalants, and heroin, but not greater alcohol-related problems (AUDIT-C), or increased past 30-day use of alcohol, cannabis (medical or recreational), hallucinogens, amphetamines, methamphetamines, ecstasy or nonmedical use of any prescription drugs. Furthermore, meeting key criteria was not significantly associated with receiving past treatment for substance use. Meeting a greater number of criteria was associated with significantly greater alcohol-related problems and greater likelihoods of past 30-day use of cocaine, inhalants, amphetamines, methamphetamines, and nonmedical use of prescription stimulants, opioids, tranquilizers, and sedatives, but not past 30-day use of alcohol, cannabis, hallucinogens, or ecstasy. Meeting a greater number of criteria was associated with a significantly greater likelihood of a history of substance use treatment.

## Discussion

This study provides novel information about the prevalence and correlates of meeting diagnostic criteria for caffeine use disorder among a diverse sample of U.S. adults as well as the relationship between meeting diagnostic criteria and caffeine-related functional impairment. Overall, 8% of the sample met the three *DSM*-proposed key criteria for caffeine use disorder. Individuals who met key criteria demonstrated significantly greater overall distress related to their caffeine consumption relative to individuals who did not meet criteria. Furthermore, our analyses showed an orderly relationship between the total number of criteria met and caffeine-related distress and functional impairment. This suggests that the number of criteria met might be a useful index of severity of caffeine use disorder above and beyond key criteria. Moreover, the present study found that individuals who consumed more caffeine, cigarette smokers, and younger individuals were more likely to meet criteria for caffeine use disorder. Finally, individuals meeting criteria had significantly worse sleep and significantly greater depression, anxiety, and stress relative to individuals who did not meet criteria after controlling for smoking and demographic variables. This information will be important when caffeine use disorder is considered for inclusion in the future *DSM* and in national epidemiological research.

The observed prevalence of caffeine use disorder symptoms in the present study is generally consistent with prior estimates of prevalence of meeting criteria roughly similar to the three key diagnostic criteria, which range from less than 10% to 13% among general samples of adults living in the U.S., Italy, and Hungary.^6,22,27^ The prevalence of caffeine use disorder might be higher among special populations. Among patients seeking treatment for problematic caffeine consumption, the prevalence of meeting all three key diagnostic criteria for caffeine use disorder ranges from 72% to 84%.^24,26,32^ Other studies examining the prevalence of caffeine dependence broadly defined among samples such as heavy caffeine consumers; or young adults, college students, adolescents, and adults with a history of drug use indicate potentially higher estimates of problematic caffeine consumption (e.g., 20% or greater).^16–19,21–23,25^ Our data also support that meeting a minimum of at least two criteria, the threshold used for mild substance use disorder, might be inappropriate for caffeine use disorder. Nearly half of our sample met at least two criteria, which suggests this lower threshold could decrease the meaningfulness of the diagnosis. Given the ubiquity of caffeine consumption, it will be important to collect additional data regarding the informative value of specific criteria,^27^ and to develop guidelines for clinicians to carefully consider the frequency, intensity, and functional impairment associated with *DSM*-defined criteria to prevent overdiagnosis.

These data have limitations. We achieved substantial diversity in age, sex, race, and ethnicity, but the present data are not representative of the general United States population. Specifically, women, older adults, and cigarette smokers were somewhat overrepresented, whereas Hispanic individuals were somewhat underrepresented.^36,37^ Large-scale epidemiological studies such as the National Survey on Drug Use and Health should consider including *DSM-5* criteria for caffeine use disorder to determine the generality of the present results to a truly representative sample. Furthermore, we considered only whether individuals met the proposed diagnostic criteria during the past 12 months. Lifetime prevalence is likely greater. Longitudinal studies will be necessary to examine the persistence of caffeine use disorder symptoms and functional impairment over time. To maintain brevity, we completed only short self-reported assessments of sleep, anxiety, depression, stress, and substance use. These assessments did not specify the role of caffeine and could be capturing caffeine-exacerbated sleep problems or psychological symptoms, but could also be capturing *a priori* differences in sleep and psychological function that preceded problematic caffeine consumption. Our substance use data were somewhat equivocal, possibly because of the low the overall prevalence of illicit drug and nonmedical prescription drug use. A larger sample or a sample with greater substance use history might be necessary to reliably detect the association between caffeine use disorder and other substance use. Although our data suggest potential psychological distress correlates and shared risk factors, more detailed clinical evaluation of these variables is necessary, and special examination of caffeine use disorder symptoms among individuals with anxiety disorders and sleep problems is warranted.

## Conclusion

This study is the most thorough evaluation to date of the prevalence, clinical significance, and correlates of meeting proposed criteria for caffeine use disorder. Collectively, the observed associations should inform future research and considerations regarding risk and differential diagnosis. For example, these data suggest that researchers should control for the effects of cigarette smoking and age when examining caffeine use disorder, and differential diagnosis for caffeine use disorder should include anxiety and sleep disorders. These data also illuminate potential psychological and demographic correlates, although additional work is needed among nationally representative samples and special clinical populations. These data support the inclusion of caffeine use disorder in future iterations of the *DSM*, given that only a modest percentage of a nonclinical sample of caffeine consumers met the proposed key diagnostic criteria, and meeting *DSM*-defined criteria was associated with clinically meaningful effects.
